# Feeding difficulties, food intake, and growth in children with esophageal atresia

**DOI:** 10.1002/jpr3.12136

**Published:** 2024-10-17

**Authors:** Kjersti Birketvedt, Audun Mikkelsen, Ragnhild Hanssen, Helle Schiørbeck, Hanneke IJsselstijn, Christine Henriksen, Ragnhild Emblem

**Affiliations:** ^1^ Centre of Rare Diseases, Division of Pediatric and Adolescent Medicine Oslo University Hospital Oslo Norway; ^2^ Division of Pediatric Surgery Oslo University Hospital Oslo Norway; ^3^ Department of Nutrition, Faculty of Medicine University of Oslo Oslo Norway; ^4^ Department of Pediatric Neurology Oslo University Hospital Oslo Norway; ^5^ Department of Pediatric Surgery Erasmus MC‐ Sophia Children's Hospital Rotterdam The Netherlands; ^6^ Faculty of Medicine University of Oslo Oslo Norway

**Keywords:** dietary survey, feeding development, Montreal Childrens' Hospital Feeding scale (MCH‐FS), nutritional intake, parent‐reported

## Abstract

**Objectives:**

Challenges regarding feeding difficulties and nutrition in children with esophageal atresia (EA) have been sparsely studied. The aim of this study was to explore parent‐reported feeding difficulties in children with EA by applying Montreal Children's Hospital‐Feeding Scale (MCH‐FS), and to further explore associations between feeding difficulties and clinical factors, growth and nutritional intake.

**Methods:**

Parents of EA children born between 2012 and 2017 were invited. Clinical data were collected from medical records. In a prospective cohort‐study parent‐reported feeding difficulties (by MCH‐FS) were reported at two assessments, and at the second assessment, dietary data were collected by using the 24‐h food‐recall method.

**Results:**

Out of 55 eligible participants, we evaluated 53 children at median age of 1.6 years (Q1:Q3 1.0:2.9) (first assessment) and 38 at median age of 4.2 years (Q1:Q3 1.0:2.9) (second assessment). Feeding difficulties were reported by 34% and 31% of the parents, respectively, but no particular profile of concerns could be identified. Children's energy intake and weight‐for‐age were correlated with feeding difficulties (MCH‐FS total score) (*p* < 0.02).

**Conclusion:**

Parent‐reported feeding difficulties were identified in one‐third of children with EA and related to low energy intake and low weight‐for‐age, but not to clinical factors. This implies that feeding difficulties must be screened for during follow‐up in all EA children and may facilitate early detection of challenges and intervention if needed.

## Abstract (highlights)

**What is Known:**

- Deviant feeding development and feeding difficulties have been described in children with esophageal atresia.
- Children with esophageal atresia have impaired growth in infancy and childhood.

**What is New:**

- Parent‐reported feeding difficulties as reported by Montreal Children's Hospital Feeding Scale (MCH‐FS) appeared in one‐third of children with esophageal atresia.
- Feeding difficulties as reported by MCH‐FS are associated with low energy intake and low weight‐for‐age *z*‐scores.
- The pattern of feeding difficulties varies among children with esophageal atresia, which indicates need for individual feeding and nutritional guidance.

## 1 INTRODUCTION

With improvements in survival rates for patients born with esophageal atresia (EA), emphasis has shifted towards morbidity and quality‐of‐life.^1^, ^2^, ^3^ Esophageal dysmotility, affecting most of the patients, may impact feeding and growth, especially during the first years of life.^4^, ^5^, ^6^, ^7^, ^8^

Feeding difficulties may entail nutritional deviation and significant distress and burden for children and their parents.^9^, ^10^, ^11^ International guidelines recommend health professionals to recognize and identify early symptoms of feeding difficulties in children with EA.^2^, ^3^ However, relations between various feeding difficulties and dietary composition, growth and comorbidity have not been widely explored and validated tools are missing.

In 2019, Goday et al. defined pediatric feeding disorders (PFD) as “impaired, not age‐appropriate oral intake associated with medical, nutritional, feeding skills and/or psychosocial dysfunction.”^12^ Identifying feeding difficulties in a structured way would allow for earlier targeted interventions and support.^9^, ^10^, ^13^ Recently, the Montreal Children's Hospital Feeding scale (MCH‐FS) has been recommended for identifying feeding difficulties in children,^14^ and has also been found appropriate in the care of EA patients.^15^, ^16^, ^17^

The primary aim of this study was to explore parent‐reported feeding difficulties in young children with EA by applying MCH‐FS. The secondary aim was to explore associations between feeding difficulties, clinical factors, growth and nutritional intake.

## 2 MATERIALS AND METHODS

### 2.1 Participants

Fifty‐seven EA patients were born during the period of 2012–2017, two died neonatally. The first assessment in the study was performed during regular follow‐up visits between 2016 and 2018, and the second assessment was performed in 2018. The study was approved by the Regional Ethical Committee in Norway, reg. no: 2014/1224.

### 2.2 Design

Using a prospective cohort design, parent‐reported feeding difficulties and growth data were recorded at the two assessments. During the second assessment parents were invited to take part in an additional dietary survey and semi‐structured interview regarding feeding history and eating habits.

### 2.3 Clinical data

Medical records were reviewed for EA classification according to Gross classification,^18^ associated anomalies, prematurity (gestational age <37 weeks), length of postnatal hospital stay, duration of tube feeding, history of gastrostomy (ever been tube‐fed by gastrostomy), and esophageal dilations due to stricture. The vertebral defects, anal atresia, cardiac, trachea, esophageal, renal, and limb defects syndrome (VACTERL) was defined according to Solomon.^19^

### 2.4 Feeding difficulties

To identify feeding difficulties we used MCH‐FS in its approved Norwegian version.^14^ The Norwegian version had been translated according to international guidelines. MCH‐FS was recorded during the first assessment at regular follow‐up in 2016–2018 and at the second assessment as a part of a diet survey in 2018. MCH‐FS is a valid and reliable screening tool consisting of 14 items covering different feeding problems in children 0.5–6 years.^14^ Parents respond to each item based on a 7‐point Likert scale (range, 1–7). The scoring sheet allows to report total items scores and categorization of feeding as “normal” = total score ≤45, “with mild difficulties” = total score 46–52, “moderate difficulties” = total score 53–58 and “severe difficulties” = total score 59–98.^14^ The reported data were analyzed according to the manual, using original Canadian normative values.^14^ Individual analyses of item scores in relation to normative values were performed in participants classified as having moderate/severe feeding difficulties.

### 2.5 Anthropometry

Physical growth was evaluated at both assessments by weight and height measurements by trained staff using standardized measuring equipment. In children <18 months body length was measured supine with ADE MZ10023‐3 and in children >2 years in standard upright position with ADE MZ10023‐3 to the nearest 0.1 cm. Body weight in infants was measured on a digital scale (Body‐Scale‐Model, Solotop OY) to the nearest 0.005 kg and with Coline for children >1 year.

Sex‐ and age‐adjusted *Z*‐scores were calculated for height (height‐for‐age *Z*‐score [HAZ]), weight (weight‐for‐age *Z*‐score [WAZ]), weight related to height for children below 2 years (weight‐for‐height *Z*‐score [WHZ]) and body mass index *Z*‐score (BMI‐Z) for children above 2 years, by using the Norwegian national reference standards and a growth analyzer calculation tool (Vekstjournalen).^20^ According to existing guidelines, *Z*‐scores were corrected for prematurity until the age of 2 years. To calculate birth weight data for preterm newborn, we used the 2013 FENTON growth charts.^21^ According to the World Health Organization, stunting was defined as HAZ < −2, underweight as WAZ < −2 and severely underweight as WAZ < −3, wasting as WHZ <−2 (children < 2 years) and BMI‐Z < −2.0 (children >2 years). Overweight was defined as BMI‐Z > 1 and obesity as BMI‐Z > 2.^22^, ^23^

### 2.6 Dietary survey

Within the second assessment we performed a dietary survey to collect food‐intake data by a structured 24‐h food‐recall.^24^, ^25^ Before the interview a registration form and a validated booklet of photographs of foods commonly eaten by Norwegian children were sent to participants.^26^ We performed two 24‐h recalls per child, from a weekday and a weekend‐day. The telephone interviews were performed by a dietitian student, supervised by a registered dietitian. The data on food intake was entered into an online Norwegian nutrition analysis tool (Kostholdsplanleggeren).^27^ National Nutrition Recommendations 2012 was used as reference data.^28^, ^29^

### 2.7 Semi‐structured interview

Semi‐structured telephone interview was part of the dietary investigation and was performed by the dietitian student at the same time as the 24‐h food‐recall. The interviews‐manual was developed for this study by experienced dietitian with specific knowledge about children with esophageal atresia and included standardized questions regarding the child's feeding history and eating habits.

### 2.8 Statistics

Data from clinical assessments, growth parameters and dietary factors were described in terms of prevalence (*n*, %), and as mean (SD) or median (Q1:Q3) according to the results of normality test supported by Q‐Q plot in SPSS. Differences between groups in first and second assessment were analyzed using Student *t*‐test for Gaussian distributed variables and Mann–Whitney *U*‐test elsewhere. Categorical variables were compared by Chi square test, for example, for participants with and without feeding difficulties (Table S1). Independence was calculated with Pearson correlation coefficient and Spearman's rho for nutritional parameters versus MCH‐FS total score in both assessments. Analyses of energy intake and WAZ in relation to feeding difficulties (MCH‐FS total score) was performed using parametric correlation analysis. Significance level was <0.05. Statistical analysis were performed using SPSS software version 28.0 (IBM).

## 3 RESULTS

### 3.1 Study population

**Figure 1:**
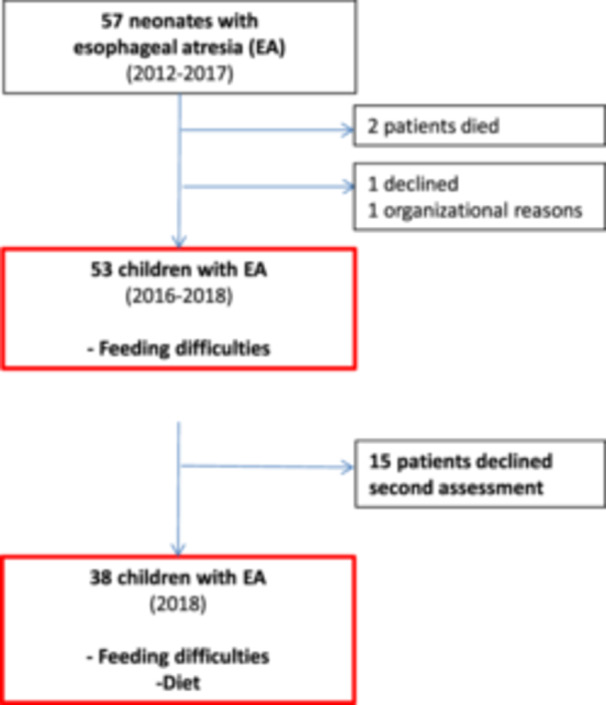
Participants in the study of children with esophageal atresia.

Out of 55 eligible patients, 2 were excluded. The parents of the remaining 53 patients (96%) accepted the invitation to the first assessment of parent‐reported feeding difficulties and growth (Figure 1). Parents of 38 patients (69%) accepted the invitation to a second assessment including the dietary survey. These 38 patients did not differ from the 15 declining second assessment in clinical factors and MCH‐FS score in first assessment. Median interval between first and second assessment was 2.5 years (1.5:3.0).

### 3.2 Clinical data

Demographics and clinical data of the participants are reported in Table 1. Median age at the two assessments, was 1.7 years (1.0:2.9) and 4.2 years (2.2:5.7), respectively.

**Table 1:** Demographics, clinical and feeding data at first and second assessment.

	Participants in first assessment *n* = 53	Participants in second assessment *n* = 38
Male, *n* (%)	30 (57)	22 (58)
Age at first assessment (year), median (Q1:Q3)	1.6 (1.0:2.9)	4.2 (2.2:5.7)
Birth weight (g), median (Q1:Q3)	2630 (2100:3160)	2665 (2152:3232)
Prematurity (GA < 37 weeks), *n* (%)	23 (43)	16 (42)
Gross, type C, *n* (%)	47 (89)	35 (92)
Associated anomalies, *n*(%)	25 (47)	19 (50)
Cardiovascular anomalies, *n* (%)	17 (32)	12 (32)
VACTERL‐association, *n* (%)	14 (26)	10 (26)
Esophageal dilatations <12 months, median (Q1:Q3)	2 (0:7)	1 (0:7)
More than three esophageal dilatations, *n* (%)	20 (38)	14 (37)
Tube feeding after birth (days), median (Q1:Q3)	20 (11:49)	18 (11:45)
History of gastrostomy, *n* (%)^a^	11 (21)	8 (21)
MCH‐FS total score, mean (SD)	39 (12)	38 (12)*
Normal feeding, *n* (%)	35 (66)	24 (69)*
Feeding difficulties, *n* (%)	18 (34)	11 (31)
Mild feeding difficulties, *n* (%)	9 (17)	6 (17)*
Moderate feeding difficulties, *n* (%)	8 (15)	4 (11)*
Severe feeding difficulties, *n* (%)	1 (2)	1 (3)*

Abbreviations: GA, gestational age; MCH‐FS, Montreal Children's Hospital‐Feeding Scale; SD, standard deviation; VACTERL vertebral defects, anal atresia, cardiac, trachea, esophageal, renal, and limb defects syndrome.

^a^ One patient was partial tube‐feeding by gastrostomy at both assessments (Gross type C, VACTERL association, preterm).

* 35 participants answered MCH‐FS in the second assessment.

### 3.3 Parent‐reported feeding difficulties

Feeding difficulties, of different severity, were reported by one‐third of the parents, 34% and 31% in first and second assessment, respectively (Table 1). However, feeding difficulties were classified as “severe” only once at each assessment (different individuals). The total score did not differ significantly between participants in the first and second assessment. The child needing partial tube‐feeding, was reported to have “mild feeding difficulties” at both assessments.

**Figure 2:**
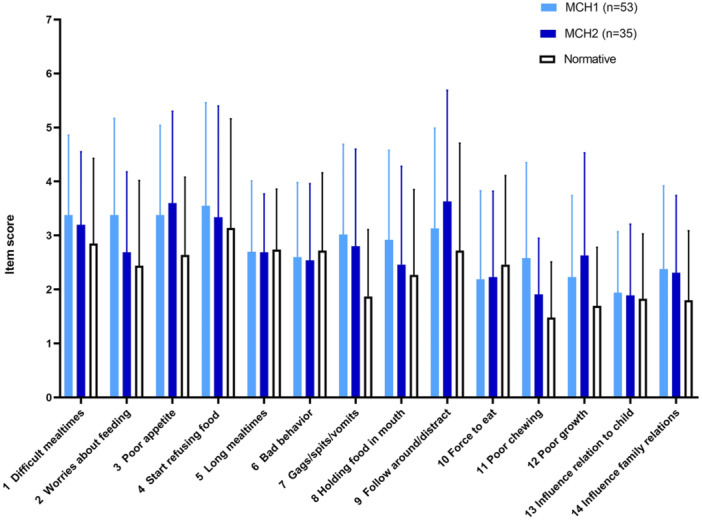
MCH‐FS item scores at two assessments and normative values, mean (SD). MCH‐FS, **M**ontreal **C**hildren's **H**ospital **F**eeding **S**cale (14). MCH1 = first assessment, MCH2 = second assessment. Parents responded on a Likert scale (range, 1–7) for each item.

Looking at the item scores of all 14 questions, mean item scores did not differ significantly from the normative data (Figure 2). Notably, the mean scores for long mealtimes (item 5) were similar in the study participants and normative population. However, we noted a large variation for each item in both participants and normative data. Next, we evaluated the 12 children, 7 in the first and 5 in the second assessment, classified as “moderate” or “severe feeding difficulties.” We did not find any specific pattern in the responses on MCH‐FS items in children with feeding difficulties, as illustrated by the five children in second as sessment in Figure S1.

In the semi‐structured interview (*n* = 36), 53% of the children were breastfed during infancy and 69% ate textures as expected for age (at present). Regarding eating habits, 92% of the parents had experiences of foods getting stuck in the child's throat, 31% reported the need of extra liquid to ensure adequate swallowing and 23% reported symptoms of feeding being burdensome. According to the interview‐data, 61% had had counseling by a dietitian (interview‐data is presented in Table S2). Parent‐reported feeding difficulties in the first and second assessment are presented by age‐groups in Table 2. Children 1–2 years of age appeared to be more challenging, but the sample size is insufficient for statistical analysis.

**Table 2:** Parent‐reported feeding problems at two assessments (MCH‐FS 1 and MCH‐FS 2), by age groups.

	MCH‐FS 1 (*n* = 53)				MCH‐FS 2 (*n* = 35)			
Age groups (year)	0.5–1	1–2	2–4	>4	0.5–1	1–2	2–4	>4
*n*	11	18	20	4	0	7	9	19
Total raw‐score, mean (SD)	37 (15.6)	43 (9.4)	39 (11.8)	33 (5.2)	‐	47 (9.4)	35 (13.2)	36 (11.8)
Normal feeding, *n* (%)	8/11 (73)	10/18 (56)	13/20 (65)	4/4 (100)	‐	3/7 (43)	7/9 (78)	14/19 (74)
Any feeding difficulties, *n* (%)	3/11 (27)	8/19 (44)	7/20 (35)	‐	‐	4/7 (57)	2/9 (22)	5/19 (26)

*Note*: Total score (raw score) < 45 indicate “normal feeding.”

### 3.4 Anthropometry

Overall, more than 80% of the participants were categorized as having normal HAZ, WAZ, at both assessments (Table S3). Longitudinal growth data from the regular follow‐up shows that height and weight impairment were reduced from the age of 6 to the age of 48 months (Table S4).

### 3.5 Nutritional intake

According to the 24‐h food‐recall, parent‐reported energy and macronutrient intake were in the mid or in the upper range of the recommendations for all age groups^28^, ^29^ (Table S5). However, intake of saturated fat expressed as percent of total energy intake was 14.2 (1–2 years), and 16.3 (2–4 years) which is above recommended intake.

### 3.6 Factors associated with feeding difficulties

To explore factors influencing feeding difficulties, we analyzed clinical data and compared children with “normal feeding” and “feeding difficulties” (all categories), Table S1. Background characteristics did not differ between those with and without feeding difficulties, apart from WAZ being lower in children with proxy‐reported difficulties in the first assessment (*p* = 0.007). The two children born with long gap EA did not exhibit any typical characteristics concerning feeding.

Correlation analysis showed that WAZ was correlated to feeding difficulties (MCH‐FS total score) at both assessments correlation coefficients −0.356 and −0.378; *p* = 0.014 and 0.010, respectively. Furthermore, children's energy intake was associated with feeding difficulties (correlation coefficient −0.376; *p* = 0.011), but here was no correlation between the intake of saturated fat and parent‐reported feeding difficulties.

## 4 DISCUSSION

In this prospective cohort‐study, feeding difficulties as assessed by the MCH‐FS, were identified in one‐third of children with EA. Parental reported feeding difficulties were mostly recorded in children with low energy intake and low weight‐for‐age (WAZ). The pattern of feeding difficulties varies, and we were unable to identify a particular pattern characterizing challenges in children with EA.

In our study, one‐third of the children had feeding difficulties according to parental reports. This prevalence aligns with the wide range of prevalence (26%–79%) presented in previous research, that have estimated feeding challenges from symptoms of feeding and swallowing difficulties in patients with EA.^30^, ^31^, ^32^, ^33^ Recently, MCH‐FS has been employed to evaluate children with EA.^15^, ^16^ Pham et al. presenting data of 145 children with a median age of 2.3 years (1.8:2.9), report one‐third with feeding difficulties and 9.5% with severe feeding difficulties, which seems to be in accordance with our results.^16^ However, 19 patients (13%) in Pham's EA population were tube‐fed and excluded from participation. In our study, the parents of the partly gastrostomy fed child reported only mild feeding difficulties at both assessments. We assume that insertion of a gastrostomy tube may reduce parental stress and hence promote a better oral intake and improved parent–child communication, which could be evaluated by the MCH‐FS.^34^, ^35^

In our study population the energy intake was within normal range for age groups. However, low energy intake was correlated to MCH‐score, indicating that the parents whose children had low energy intake were the ones reporting most feeding difficulties. Furthermore, high scores on feeding difficulties were also associated with low weight for age. The correlation between MCH‐FS and energy intake and WAZ may add knowledge to the background for impaired growth in children with EA. Measuring energy intake in children is challenging, but parents reporting feeding difficulties as assessed by MCH‐FS may considered as a warning sign for low energy that warrants further evaluation by a dietitian.

Surprisingly, the intake of saturated fat was above the recommendations,^28^, ^29^ but not correlated to parental reported feeding difficulties. The high proportion of energy from saturated fats in the diet of children with EA has previously been described by Traini.^36^ To serve a more high‐fat diet to children with EA is in line with a previous study describing that parents serve “an energy‐enriched diet” to their child, especially in ages 2–7 years.^37^ Fitzgerald et al. advocated some feeding strategies to improve energy intake and emphasize that enrichment with carbohydrates and/or fat alone may not be sufficient, as this will result in a reduced energy ratio to the feed and may hinder the potential for optimal weight gain.^38^

Even if insufficient energy intake obviously is one of the important reasons for impaired growth, there is a lack of knowledge of the relationship between diet and impairments in growth in children with EA.^39^ Based on our findings, it may be interesting to explore growth factors in relation to diet and feeding skills in future studies in children with EA.

We observed a broad variation in the individual responses to the 14 questions in the MCH‐FS screening tool. This means we cannot elaborate any typical EA feeding challenges, nor in the participants with most serious feeding difficulties. However, since there is no disease‐typical patterns of items or factors describing feeding difficulties in EA individual evaluation and counseling are necessary for children with high scores on parental reports.

No clinical factors were associated with feeding difficulties is the present study. This is in contrast to Baird et al. including 30 caregivers of children with EA summarizing that patients with complicated EA (non‐type C and prematurity) had significantly higher MCH‐FS scores than those with EA type C.^15^ On the other hand, Pham's results support our findings describing no association between prematurity, associated congenital malformations, type of EA, anastomotic complications and feeding difficulties in children with EA, meaning that all children with EA may develop feeding difficulties.^16^

Parents of children with EA seemed to experience more burdensome feeding in children ages 1–2 years. This can be interpreted and supported by previous studies, describing the weaning period as the most stressful.^40^, ^41^ Furthermore, Harrington et al. presented 19% (8/43) of EA children at 1 year needed “special adaptions of the food,” compared to 9% and 0 at ages 2 and 3 years.^42^ However, as we know no studies using MCH‐FS have described differences between age groups. Thus, infants and toddlers with EA should be offered individualized and tailored feeding‐ and nutrition counseling during follow‐up.

Among strengths of our study is that all EA children during follow‐up at a specialist clinic in Norway were included. Furthermore, the study has a high participation rate, 92% and 67% in the first and second assessment, respectively. Feeding difficulties and nutrient intake were mapped and evaluated by well‐established methods. Furthermore, MCH‐FS is a validated and recognized questionnaire.

Clinical research in rare diseases presents several challenges of which sample size is among the most important. The relatively small sample size that was examined in the present study may affect the results and is the main limitation to the study. Therefore, our data set did not allow for further analysis of risk factors such as long gap EA or gastrostomy tube‐feeding. Furthermore, the participants had a large age range. Considering the current definition of feeding difficulties, the child's age is crucial.^12^ MCH‐FS not discriminating between age groups and may be a somewhat rough screening tool.^10^ Furthermore, we did not have a control group of healthy children, and the Canadian normative data do not distinguish age‐specific scores. The interval between the two assessments varied, and we could not assess individual changes between the two assessments. The semi‐structured interview was developed for this study and was not based on a validated tool. Furthermore, we did not record parental factors. MCH‐FS does not consider the parents health literacy, which may influence parental scoring of feeding challenges. MCH‐FS was developed on a biopsychological model and report feeding difficulties from the parent's perspective, with little focus on the functional oral intake. Further research with instruments covering more aspects of the whole feeding process is necessary.

## 5 CONCLUSION

Parent‐reported feeding difficulties are frequent in children with EA and associated with low energy intake and inadequate weight gain. Thus, screening for parent‐reported feeding difficulties may help identifying patients requiring nutritional assessment and intervention.

## CONFLICT OF INTEREST STATEMENT

The authors declare no conflict of interest.

## Supporting information

Supporting information.

Supporting information.

Supporting information.

Supporting information.

Supporting information.

Supporting information.
