# Supplementary material for: Feeding difficulties, food intake, and growth in children with esophageal atresia
Source: JPGN Rep. 2024 Oct 17;5(4):462–9. doi: 10.1002/jpr3.12136 (PMC11600379; doi:10.1002/jpr3.12136)
Supplement: Supplementary file 1 — Supporting information. [file JPR3-5-462-s006.docx]

**Supplementary figure 1. Individual items score in patients categorized with moderate/severe feeding difficulties in second assessment (n=5)**
**
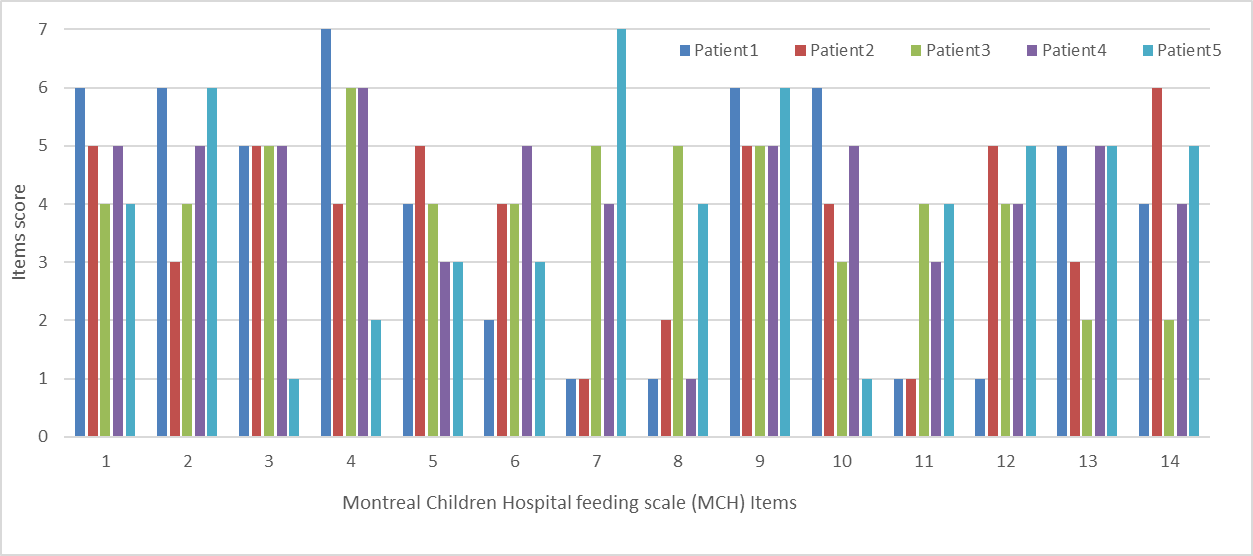
**
