# Supplementary material for: Feeding difficulties, food intake, and growth in children with esophageal atresia
Source: JPGN Rep. 2024 Oct 17;5(4):462–9. doi: 10.1002/jpr3.12136 (PMC11600379; doi:10.1002/jpr3.12136)
Supplement: Supplementary file 2 — Supporting information. [file JPR3-5-462-s001.docx]

**Supplementary table 1. Characteristics in patients with normal feeding and feeding difficulties**

|  | First assessment  n=53 | | p | Second assessment  n=38* | | p |
| --- | --- | --- | --- | --- | --- | --- |
|  | **Normal feeding**** | **Feeding difficulties***** |  | **Normal feeding**** | **Feeding difficulties***** |  |
|  | n=35 | n=18 |  | n=24 | n=11 |  |
| Male, n (%) | 20/35 (57) | 11/18 (61) | 0.781 | 15/24 (63) | 6/11 (55) | 0.656 |
| Age, mean (SD) | 2.1 (1.2) | 1.8 (1.1) | 0.436 | 4.1 (1.8) | 3.9 (2.1) | 0.708 |
| Prematurity (GA < 37 weeks), n (%) | 15 (43) | 8 (44) | 0.912 | 9 (38) | 5 (45) | 0.656 |
| Birth weight (g), mean (SD) | 2586 (820) | 2548 (655) | 0.864 | 2761 (740) | 2386 (590) | 0.150 |
| Gross, type C, n (%) | 30 (86) | 17 (94) | 0.342 | 22 (92) | 11 (100) | 0.324 |
| Cardiovascular anomalies, n (%) | 9 (26) | 8 (44) | 0.167 | 6 (25) | 5 (45) | 0.226 |
| Symptoms of GERD ****, n (%) | 9 (26) | 10 (56) | 0.068 | 5 (21) | 9 (82) | 0.002 |
| VACTERL-association, n (%) | 7 (20) | 7 (39) | 0.140 | 5 (21) | 4 (36) | 0.329 |
| Esophageal dilatations < 12 mo, median (Q1:Q3) | 1 (0:6) | 2 (1:9) | 0.299 | 1 (0:6) | 2 (0:8) | 0.635 |
| More than 3 esophageal dilatations, n (%) | 12 (34) | 8 (44) | 0.630 | 8 (33) | 4 (36) | 0.923 |
| Neonatal tube feeding (days), median (Q1:Q3) | 17 (13:139) | 51 (10:53) | 0.171 | 14 (11:28) | 20 (13:45) | 0.313 |
| History of gastrostomy, n (%) | 5 (14) | 6 (33) | 0.105 | 3 (13) | 3 (27) | 0.282 |
| Height for age z-score (HAZ), mean (SD) | -0.4 (1.2) | -0.9 (1.0) | 0.099 | -0.4 (1.2) | -1.0 (1.3) | 0.186 |
| Weight for age z-score WAZ , mean (SD) | -0.7 (0.9) | -1.4 (0.9) | 0.007 | -0.8 (1.0) | -1.3 (0.83) | 0.130 |

* 35/38 participants answered MCH-FS in second assessment
**Normal feeding according to Montreal Children Hospital Feeding Scale (MCH-FS) with raw score <45
***Feeding Difficulties according to Montreal Children Hospital Feeding Scale (MCH-FS) with raw score ≥ 46
****Symptoms of gastroesophageal reflux disease (GERD) according to medical records
