# Supplementary material for: Feeding difficulties, food intake, and growth in children with esophageal atresia
Source: JPGN Rep. 2024 Oct 17;5(4):462–9. doi: 10.1002/jpr3.12136 (PMC11600379; doi:10.1002/jpr3.12136)
Supplement: Supplementary file 3 — Supporting information. [file JPR3-5-462-s004.docx]

**Supplementary table 2. Parents’ answers in the interview at second assessment (n=36)**

| Questions in semi-structured interview | Answering “yes”,  number (%) |
| --- | --- |
| Breastfeeding in infancy | 19 (53 %) |
| The child eats textures as expected for age (at present) | 25 (69 %) |
| The child finds feeding burdensome | 8 (23 %) |
| Need of extra liquid to ensure swallowing | 11 (31 %) |
| Ever experiences of foods getting stuck in the child’s throat | 33 (92 %) |
| Counselling by dietitian (any time in follow-up) | 22 (61 %) |
