# Supplementary material for: Feeding difficulties, food intake, and growth in children with esophageal atresia
Source: JPGN Rep. 2024 Oct 17;5(4):462–9. doi: 10.1002/jpr3.12136 (PMC11600379; doi:10.1002/jpr3.12136)
Supplement: Supplementary file 4 — Supporting information. [file JPR3-5-462-s003.docx]

**Supplementary table 3. Growth data at first and second assessment**

|  | First assessment | Second assessment |
| --- | --- | --- |
| Weight for age z-score (WAZ) z-score, median (Q1:Q3) normal, n (%) underweight, n (%) severely underweight, n (%) | n=53  -0.88 (-1.72:-0.27) 47 (89) 5 (9) 1(2) | n=37^a^  -0.90 (-1.57:-0.31) 31 (84) 5 (14) 1 (3) |
| Height for age z-score (HAZ) z-score, median (Q1:Q3) normal height, n (%) stunted, n (%) | n=53  -0.53 (-1.39:0.19) 48 (91) 5 (9) | n=36^a^  -0.40 (-1.48:0.34) 32 (86) 4 (11) |

*^a^Missing growth-data caused by organizational reasons*
