# Supplementary material for: Feeding difficulties, food intake, and growth in children with esophageal atresia
Source: JPGN Rep. 2024 Oct 17;5(4):462–9. doi: 10.1002/jpr3.12136 (PMC11600379; doi:10.1002/jpr3.12136)
Supplement: Supplementary file 5 — Supporting information. [file JPR3-5-462-s005.docx]

**Supplementary table 4. Follow-up growth data in children participated in both assessments (n=38)**

| Growth variables | Age | | | | | |
| --- | --- | --- | --- | --- | --- | --- |
| Age category (months) | 0^1^ | 6 | 12 | 24 | 48 | 6 years |
| n | 38 | 38 | 38 | 38 | 30 | 11 |
| Age (year), mean (SD) |  | 0.53 (0.07) | 1.06 (0.11) | 2.06 (0.13) | 4.08 (0.19) | 5.6 (1.0) |
| Weight (kg), mean (SD) | 2.6 (0.7) | 6.7 (1.1) | 8.8 (1.1) | 11.4 (1.3) | 15.7 (1.8) | 19.0 (1.5) |
| Height (cm), mean (SD) | 47 (4.1) | 64 (3.7) | 74 (3.9) | 85.6(4.2) | 102.4(5.1) | 116.2 (5.2) |
| Height for age z-score (HAZ)   z-score, mean (SD)  normal, n (%)  < -2 (stunted), n (%) | -1.7(2.0) 24 (63) 14 (37) | -1.5(1.4)  25 (66) 13 (34) | -1.0(1.2)  32 **(84)**  6 (16) | -0.7 (1.4) 32 **(84)**  6 (16) | -0.5 (1.2) 26 **(87)**  4 (13) | -0.3 (0.1) 11 **(100)** - |
| Weight for age z-score (WAZ)  z-score, mean (SD)  normal, n (%)  <-2 (underweight), n (%) | -2.4 (1.7)  20 (53) 18 (47) | -1.6 (1.3)  26 (68) 12 (32) | -1.2 (1.1)  29 (76)  9 (24) | -1.0 (1.0) 32 **(84)**  6 (16) | -0.9 (1.0) 26 **(87)**  4 (13) | -0.9 (0.7) 10 **(91)**  1 (9) |
| Weight for height z-score (WHZ)  z-score, mean (SD)  normal, n (%)  <-2 (wasted)  > 2 (overweight) |  | -0.7 (1.1)  30 (79) 7 (18) 1 (3) | -0.9 (1.1)  31 (82) 7 (18) |  |  |  |
| BMI for age z-score (BMI-Z)  z-score, mean (SD)  normal, n (%)  < -2 (wasted)  > 1 (overweight) |  |  |  | -0.7 (1.1) 34 (89)  3 (8)  1 (3) | -0.8 (1.2) 24 (80)  5 (17)  1 (3) | -1.3 (0.9)  8 (73)  3 (27) |

^1^Fenton curves
