# Supplementary material for: Feeding difficulties, food intake, and growth in children with esophageal atresia
Source: JPGN Rep. 2024 Oct 17;5(4):462–9. doi: 10.1002/jpr3.12136 (PMC11600379; doi:10.1002/jpr3.12136)
Supplement: Supplementary file 6 — Supporting information. [file JPR3-5-462-s002.docx]

**Supplementary table 5. Daily intake of energy and macronutrients according to age groups (n=38), mean (SD)**

|  | Mean (SD) daily intake and reference values according to age groups | | | | | | | |
| --- | --- | --- | --- | --- | --- | --- | --- | --- |
|  | **1-2 years  (n=8)** | | Recommendations* 1-2 years | | **2-4 years (n=10)** | Recommendations* 2-4 years | **4-7 years (n=20)** | Recommendations* 4-7 years |
| Energy intake (kcal/kg/day),  mean (SD) | **108.8 (24.4)** | | 82-89 |  | |  |  |  |
| Energy (MJ/day), mean (SD) |  |  | | | **5.9 (1.5)** | 4.5-5.9 | **6.3 (1.6)** | 5.9-7.0 |
| Total protein (% TE),  mean (SD) | **13.5 (4.2)** | | 10-15 | | **14.8 (3.7)** | 10-20 | **15.0 (2.9)** | 10-20 |
| Protein (g/kg)  mean (SD) | **3.8 (1.7)** | | 3.0-4.0 | | **4.2 (0.8)** | 3.0-4.0 | **3.3 (1.2)** | >1.5 |
| Fat, total (% TE)  mean (SD) | **40.7 (6.7)** | | 30-40 | | **39.2 (4.3)** | 25-40 | **36.0 (6.9)** | 25-40 |
| Fat, saturated (% TE)  mean (SD) | **14.2 (5.8)** | | <10 | | **16.3 (5.3)** | <10 | **14.9 (3.9)** | <10 |
| Carbohydrates,  total (% TE),  mean (SD) | **45.8 (8.1)** | | 45-60 | | **46.1 (4.4)** | 45-60 | **49.0 (7.4)** | 45-60 |
| Added sugars (% TE),  mean (SD) | **7.0 (4.7)** | | <10 | | **7.9 (6.4)** | <10 | **10.7 (5.8)** | <10 |

%TE: percent of total energy intake
*Reference values, Nordic Nutrition Recommendations (2012)
